# Functional Neuronal Cells Generated by Human Parthenogenetic Stem Cells

**DOI:** 10.1371/journal.pone.0042800

**Published:** 2012-08-06

**Authors:** Ruhel Ahmad, Wanja Wolber, Sigrid Eckardt, Philipp Koch, Jessica Schmitt, Ruslan Semechkin, Christian Geis, Manfred Heckmann, Oliver Brüstle, John K. McLaughlin, Anna-Leena Sirén, Albrecht M. Müller

**Affiliations:** 1 Institute for Medical Radiation and Cell Research (MSZ) in the Center for Experimental Molecular Medicine (ZEMM), University of Würzburg, Würzburg, Germany; 2 Department of Neurosurgery, University of Würzburg, Würzburg, Germany; 3 Center for Molecular and Human Genetics, The Research Institute at Nationwide Children's Hospital, Columbus, Ohio, United States of America; 4 Institute of Reconstructive Neurobiology, Life and Brain Center, University of Bonn and Hertie Foundation, Bonn, Germany; 5 Department of Neurology, University of Würzburg, Germany; 6 Department of Physiology, University of Würzburg, Germany; 7 International Stem Cell Corporation, Oceanside, California, United States of America; Center for Regenerative Therapies Dresden, Germany

## Abstract

Parent of origin imprints on the genome have been implicated in the regulation of neural cell type differentiation. The ability of human parthenogenetic (PG) embryonic stem cells (hpESCs) to undergo neural lineage and cell type-specific differentiation is undefined. We determined the potential of hpESCs to differentiate into various neural subtypes. Concurrently, we examined DNA methylation and expression status of imprinted genes. Under culture conditions promoting neural differentiation, hpESC-derived neural stem cells (hpNSCs) gave rise to glia and neuron-like cells that expressed subtype-specific markers and generated action potentials. Analysis of imprinting in hpESCs and in hpNSCs revealed that maternal-specific gene expression patterns and imprinting marks were generally maintained in PG cells upon differentiation. Our results demonstrate that despite the lack of a paternal genome, hpESCs generate proliferating NSCs that are capable of differentiation into physiologically functional neuron-like cells and maintain allele-specific expression of imprinted genes. Thus, hpESCs can serve as a model to study the role of maternal and paternal genomes in neural development and to better understand imprinting-associated brain diseases.

## Introduction

Due to their unlimited self-renewal and multilineage differentiation potential, human pluripotent stem cells have become key cell sources for cell differentiation research, disease modeling, drug discovery, and have potential for cell replacement strategies [1]. In human, pluripotent stem cell lines can be derived from *in vitro* cultured inner cell mass (ICM) cells of blastocyst stage embryos, producing embryonic stem cell lines (hESC) [2]. More recently, factor-driven reprogramming of somatic cells has provided a long-sought strategy to generate patient- and disease-specific pluripotent stem cells, termed induced pluripotent (iPS) cells [3]. Pluripotent stem cell types, i.e. ES and iPS cells, and different lines of the same type exhibit considerable variations in respect to epigenetic status, gene expression profiles, and differentiation propensity, preventing generalized approaches but allowing for the correlation of gene expression patterns with differentiation propensities [4]. One specific ESC type that is unique in this respect is parthenogenetic (PG) ESCs that are derived from blastocysts resulting from the activation and subsequent development of an unfertilized oocyte. While asexual development of offspring from an oocyte without male genetic contribution (parthenogenesis) occurs naturally in various invertebrate and some vertebrate species [5], mammalian uniparental (PG, gynogenetic: GG, or androgenetic: AG, with only paternally derived genomes) embryos do not develop to term as a consequence of imbalanced expression of imprinted genes with parent of origin-dependent allele-specific expression patterns [6]. Despite this developmental limitation, stable ESC lines can be isolated from uniparental blastocysts of several species including human [7]–[10]. The *in vitro* differentiation capacity of murine uniparental ESC into various cell lineages, including neural and transplantable hematopoietic progenitors [11]–[16] indicates that these cells represent a unique model system to study the role of maternal and paternal genomes in normal development and the contribution of imprinting in disease development.

Paternally and maternally inherited alleles play non-redundant and reciprocal roles in brain development and plasticity [17]. Studies of the developmental capacity of murine PG and AG ICM cells following aggregation with biparental embryos revealed that PG cells preferentially seeded to the neocortex, striatum and hippocampus while AG cells contributed to hypothalamus and septum but were not found in the cortex [18]. Recent high-resolution screens in the mouse suggest that the developing and adult brain may be subject to complex effects of imprinting, including cell type and subregion specific effects, and temporal bias, with maternal-derived gene expression at earlier stages in the developing embryonic day 15 brain and paternal gene expression bias in both the prefrontal cortex and the hypothalamus of the adult brain [19].

*In vitro* differentiation studies have shown that hpESCs are capable of generating multiple cell lineages including mesenchymal stem cells, hepatocytes, pancreatic endocrine cells, retinal pigmented epithelial and neural progenitor cells [20]–[24]. However, more detailed investigation is required to verify the differentiation capability of hpESCs, particularly the potential for neurogenesis and further differentiation into functional neural subtypes. The apparent contribution bias of PG and AG ICM cells to different structures of the developing brain, the large number of imprinted brain genes, and the existence of imprinting-associated neuropsychiatric diseases [17], [25] could indicate that hpESCs have limited neural potential. Here, we establish that hpESCs can differentiate *in vitro* via NSCs into functional neuronal cells without apparent changes in imprinting status.

## Materials and Methods

### hpESCs culture

hpESCs (HLA heterozygous cell lines LLC6P (previously referred to as phESC-3) and LLC9P, phESC-6) were previously derived and described by the International Stem Cell Corporation [10]. Cell culture was performed as described [10] with slight modifications. hpESCs were maintained on mitomycin C-treated human foreskin fibroblasts (HFFs) (ATCC-LGC Standards, Wesel, Germany) at 5% CO_2_ in a medium containing knockout-DMEM, 20% knockout serum replacement (both Gibco Invitrogen, Karlsruhe, Germany), 1% non-essential amino acids, 2 mM L-glutamine (both PAA Laboratories, Cölbe, Germany), 0.1 mM ß-mercaptoethanol (Sigma-Aldrich, Schnelldorf, Germany) and 4 ng/mL FGF2 (PeproTech, Hamburg, Germany). Cultures were passaged at a 1∶3–1∶4 split ratio every 5–7 days. Medium was changed every day. For passage of hpESCs, the medium was removed and cells were incubated with Dispase (BD Biosciences, Heidelberg, Germany). After 8–10 minutes, the reaction was stopped by centrifugation and removing supernatant. Cells were washed once with medium, resuspended in fresh medium, and replated on HFFs.

### Neural in vitro differentiation

Neural differentiation of LLC6P and LLC9P cells was performed as previously described [26] with modifications. Briefly, 4-day-old embryoid bodies (EBs) were transferred to polyornithine/laminin-coated (both Sigma-Aldrich) tissue culture dishes and cells were cultured in N2 medium containing DMEM/F12, N2 supplement (1∶100; both Gibco Invitrogen) with 10 ng/mL FGF2 (R&D Systems, Abingdon, UK). For culture of attached EBs of line LLC6P 20 ng/mL fibronectin and 20 ng/mL laminin (both Sigma-Aldrich) were added to N2 medium. After 10 days, rosette structures were mechanically isolated with a needle. Clusters where further propagated as free-floating neurospheres in N2 medium containing 10 ng/mL FGF2 for 1 to 3 days. Neurospheres were dissociated into single cells by trypsin/EDTA (PAA Laboratories) incubation for 10 minutes. NSCs were plated on polyornithine/laminin-coated cell culture dishes. Media was changed to neural stem cell medium (NSCM) containing DMEM/F12, N2 supplement (1∶100; both Gibco Invitrogen), 1.6 g/L glucose, 10 ng/mL FGF2, 10 ng/mL EGF (R&D Systems), and 1 µL/mL B27 supplement (Gibco Invitrogen). The medium was changed daily. High cell densities were essential during initial plating phases, therefore, passaging was performed at high cell densities. Cells were split at a 1∶2–1∶3 ratio using trypsin/EDTA. Trypsin was inhibited by trypsin-inhibitor (Gibco Invitrogen). Next, cells were centrifuged at 300×*g* for 5 minutes at 4°C and plated on polyornithine/laminin-coated cell culture dishes. Passage number of LLC6P hpESCs at differentiation induction was 30–45; line LLC9P cells were used at passage numbers 48–60. Terminal differentiation of hpNSCs was performed in differentiation media containing DMEM/F12 (N2 supplement; 1∶50) and Neurobasal (Gibco Invitrogen) (B27 supplement; 1∶50) mixed at 1∶1 ratio. cAMP (300 ng/mL, Sigma-Aldrich) was added to the media for 28 days. For induction of dopaminergic differentiation [27], hpNSCs were cultured in differentiation media consisting of N2 medium supplemented with 200 ng/mL SHH, 100 ng/mL FGF8b (both R&D Systems), and 160 µM ascorbic acid (Sigma-Aldrich) for 8 days. Differentiation was performed for additional 20 days in differentiation media containing BDNF (20 ng/mL), 10 ng/mL GDNF (both R&D systems), 160 µM ascorbic acid, and 0.5 mM dibutyryl-cAMP (both Sigma-Aldrich). For induction of motoneurons [28], 1 µM retinoic acid (Sigma-Aldrich) was added to NSCM for 6 days in the presence of B27 supplement (1∶50) and adding 1 µg/mL SHH from day 5. From day 7, media was changed to NSCM (without FGF2 and EGF) but with B27 (1∶50), 1 µg/mL SHH and 0.01 µM RA for another 6 days. SHH was reduced to 50 ng/mL for the following 14 days, and cells were differentiated in the presence of 20 ng/mL BDNF and 20 ng/mL GDNF in differentiation media.

### Immunostaining of cultured cells

For multicolor fluorescent imaging, a SP5 Confocal Microscope (Leica, Wetzlar, Germany) was used. Cells grown on coverslips and were fixed in 4% formaldehyde, permeabilized in 0.1% Triton-X and 0.2% gelatin (Applichem, Darmstadt, Germany) and stained with the following antibodies: mouse anti-Nestin (Abcam, Cambridge, UK), rabbit anti-Sox1 (Millipore, Billerica, MA, USA), mouse anti-Sox2 (Abcam), mouse anti-Vimentin (Abcam), mouse anti-NeuN (Millipore), mouse anti-Tuj1 (Millipore), mouse anti-MAP2 (Millipore), mouse anti-GFAP (Novocastra, Wetzlar, Germany), mouse anti-O4 (R&D systems), rabbit anti-Synapsin1 (Synaptic Systems, Göttingen, Germany), mouse anti-Tau (Synaptic Systems), rabbit anti-GABA (Sigma-Aldrich), rabbit anti-TH (Sigma-Aldrich), goat anti-HB9 (Santa Cruz Biotechnology, Heidelberg, Germany). Anti mouse Cy3, Cy5 (Millipore), anti rabbit Cy3 and Cy5 and DyLight 488 (Jackson ImmunoResearch Laboratories, West Grove, PA, USA) and anti mouse FITC (Santa Cruz biotechnology) labeled secondary antibodies were used. DAPI-Moviol was used as a mounting medium and DAPI for counterstaining of nuclei.

### Whole cell patch-clamp analysis

Cells grown on glass coverslips in differentiation media for 28 days were transferred into a recording chamber and continuously superfused with extracellular solution containing 125 mM NaCl, 25 mM NaHCO_3_, 25 mM glucose, 2.5 mM KCl, 1.25 mM NaH_2_PO_4_, 2 mM CaCl_2_, 2 mM MgCl_2_ purged by 95% CO_2_/5% O_2_). The ion channel antagonists tetraethylammonium chloride (TEA, Sigma-Aldrich, 30 mM) or tetrodotoxin (TTX, Sigma-Aldrich, 1 µM) were added to the extracellular solution when indicated. All experiments were performed at room temperature using an EPC 10 double patch clamp amplifier and pulse software (HEKA, Lambrecht, Germany). Electrodes were pulled from thick-walled borosilicate glass and filled with intracellular solution (140 mM KCl, 10 mM Hepes, 10 mM EGTA, 2 mM Na_2_ATP, 2 mM MgCl_2_) and had a resistance between 3 and 4.5 MΩ. Cells were held in whole-cell configuration at −80 mV and were discarded if the series resistance was higher than 25 MΩ at the beginning of the measurements.

### Analysis of gene expression using semi-quantitative RT-PCR

Feeder cells were depleted by repeated passages on Matrigel-coated plates. Total RNA was isolated from biparental hESCs (I3 and H9 cell lines), hESC-derived neural stem cells (hNSCs), hpESCs and hpNSCs using peqGOLD RNAPure™ (Peqlabs, Göttingen, Germany). Passage numbers of hpESCs that were used to generate hpNSCs were identical. Before cDNA generation, RNA preparations were treated with DNase I (Applied Biosystems, Darmstadt, Germany). 1 µg RNA was reverse transcribed into cDNA using M-MLV reverse transcriptase (Gibco Invitrogen). For control experiments cDNA was generated from human parthenogenetic neural crest stem cells (hpNCSCs) (isolated from differentiating hpESCs at the attached EB stage), human fetal brain (hFB, 18 weeks, female; Stratagene, Santa Clara, CA, USA) and from human adipose tissue-derived mesenchymal stromal cells (hMSCs). For amplification Taq Polymerase (HT biotechnology, Cambridge, UK) was used. PCR conditions were: 94°C, 1 min, 55°C to 62°C, 60 seconds according to the primers, 72°C, 1 min (35 to 40 cycles). GAPDH was used a control. All reactions were performed on a T3 thermocycler (Biometra, Göttingen, Germany). Primers (Eurofins MWG Operon, Ebersberg, Germany) used were: *Acta1* forward (f): 5′-CAG GGC CCG AGC CGA GAG TAG-3′, reverse (r): 5′-ATA CCG ACC ATG ACG CCC TGG TG-3′, Tm: 60°C; *En1* f: 5′-GAC TCG CAG CAG CCT CTC-3′, r: 5′-GCC TGG AAC TCC GCC TTG-3′, 55.2°C ; *FoxD3* f: 5′-CTG GAA GAG AAG GAC AGC GAC GCA-3′, r: 5′-GCT GTT CTT GGG CTT GCT CGG G-3′, 60°C; *Gapdh* f: 5′-ACG ACC CCT TCA TTG ACC TCA ACT-3′, r: 5′- ATA TTT CTC GTG GTT CAC ACC CAT-3′, 60°C; *GFAP* f: 5′-GGC ACG TGC GGG AGG CGG CC-3′, r: 5′-TCT CAT CAC ATC CTT GTG C-3′, 59°C; *HoxA1* f: 5′-GGG TGT CCT ACT CCC ACT CA-3′, r: 5′-GGA CCA TGG GAG ATG AGA GA-3′, 62.4°C; *HoxA2* f: 5′-TTC AGC AAA ATG CCC TCT CT-3′, r: 5′-TAG GCC AGC TCC ACA GTT CT-3′, 60.5°C; *Musashi1* (*MS1*) f: 5′-GTC CTG TCG CCC ACC ATC TC-3′, r: 5′-CCC TCC CAA CGC CAC TGA C-3′, 60°C; *Nanog* f: 5′-GCT TGC CTT GCT TTG AAG CA-3′, r: 5′-TTC TTG ACT GGG ACC TTG TC-3′, 57°C; *Nestin* f: 5′-AGA GGG GAA TTC CTG GAG-3′, r: 5′-CTG AGG ACC AGG ACT CTC TA-3′, 58°C; *Nurr1* f: 5′-TTC TCC TTT AAG CAA TCG CCC-3′, r: 5′-AAG CCT TTG CAG CCC TCA CAG-3′, 60°C; *Oct4* f: 5′-CGA CCA TCT GCC GCT TTG AG-3′, r: 5′-CCC CCT GTC CCC CAT TCC TA-3′, 62°C; *Olig2* f: 5′-CAG AAG CGC TGA TGG TCA TA-3′, r: 5′-TCG GCA GTT TTG GGT TAT TC-3′, 60°C; *Pax2* f: 5′-CAG GCA TCA GAG CAC AT C-3′, r: 5′-GTC ACG ACC AGT CAC AAC-3′, 55.7°C; *Pax6* f: 5′-AAT AAC CTG CCT ATG CAA CCC-3′, r: 5′-AAC TTG AAC TGG AAC TGA CAC AC-3′, 59°C; *Snai2* f: 5′-ATA CCA CAA CCA GAG ATC CTC A-3′, r: 5′-GAC TCA CTC GCC CCA AAG ATG-3′, 60°C; *Sox1* f: 5′-TAC AGC ATG TCC TAC TCG CAG-3′, r: 5′-CTC TGG ACC AAA CTG TGG CG-3′, 61°C; *S100B* f: 5′- AAA GAG CAG GAG GTT GTG G A-3′, r: 5′- AGG AAA GGT TTG GCT GCT TT-3′, 60°C; *Tuj1* f: 5′- CAA CAG CAC GGC CAT CCA GG-3′, r: 5′-CTT GGG GCC CTG GGC CTC CGA-3′, 60°C. Expression analyses of mitotic checkpoint and extracellular matrix genes by RT-PCR were performed using QuantiTect Primer Assays (Qiagen, Düsseldorf, Germany).

### Analysis of imprinted gene expression using quantitative RT-PCR

RT-PCR reactions were performed and quantified using a Rotor-Gene 3000 (Corbett Life Science, LTF Labortechnologie, Wasserburg, Germany) and ABsolute QPCR SYBR Green Mix (ABgene, Hamburg, Germany). RT-PCR conditions were: 94°C, 30 seconds, 60°C, 30 seconds and 72°C, 30 seconds (35 cycles). The relative gene expression levels were calculated with the 2^−ΔΔCt^ method. The Ct-values indicate a difference of Ct-values between reference gene and target gene. The housekeeping gene GAPDH was used as reference. The expression level of target genes in hESCs and hNSCs were set to 1 in order to determine differences of target gene expression in hpESCs and hpNSCs respectively. The primer sequences (Eurofins MWG Operon) used were: *Cdkn1c* f: 5′-TGA AGG ACC AGC CTC TCT CG-3′, r: 5′-TTC TCC TGC GCA GTT CTC TTG-3′; *Dlx5* f: 5′-CCA ACC AGC CAG AGA AAG AA-3′, r: 5′-GCA AGG CGA GGT ACT GAG TC-3′; GAPDH f: 5′-GGA GTC AAC GGA TTT GGT CG-3′, r: 5′-TCC TGG AAG ATG GTG ATG GG-3′: *Gtl2* f: 5′-ATC AGC CAA GCT TCT TGG AA-3′, r: 5′-AGC TTC CAT CCG CAG TTC T-3′; *H19* f: 5′-CGG ACA CAA AAC CCT CTA GCT TGG AAA-3′, r: 5′-GCG TAA TGG AAT GCT TGA AGG CTG CTC-3′; *Igf2* f: 5′-CTT GGA CTT TGA GTC AAA TTG G-3′, r: 5′-CCT CCT TTG GTC TTA CTG GG-3′; *Igf2r* f: 5′-CCA TTC AGA CAA CGA CGG ATA C-3′, r: 5′-ACG TTA TAT CCT TGC GAA CTG TTT AG-3′; *Kcnk9* f: 5′-CTA CTT TGC GAT CAC GGT CA-3′, r: 5′-GTA GCG CAC GAA GGT GTT C-3′; *Kcnq1* f: 5′-TGT CCA CCA TCG AGC AGT ATG-3′, r: 5′-CCG TCC CGA AGA ACA CCA C-3′; *Kcnq1ot1* f: 5′-CCA CCT TCT CCA TCT GCT CA-3′, r: 5′-AAT CCA GTG GGG AAA AGG TC-3′; *Nnat* f: 5′-AAT CAA AAC ACC GCA CCA G-3′, r: 5′-ATC AGT GAG GGG CAA GGG GGG TTC-3′; *Snrpn* f: 5′-TGG CAC CTT TAA GGC TTT TG-3′, r: 5′-CCG CTT TTC TTC ACG CTC T-3′; Ube3a f; 5′-AGC CGG AAT CTA GAT TTC CA-3′, r: 5′-TGT CTG TGC CCG TTG TAA ACT-3′.

### Bisulfite sequencing

Genomic DNA isolated from ESCs and NSCs using DNeasy Tissue Kit (Qiagen) was modified using the EZ DNA Methylation-Gold kit (Zymo Research, Irvine, CA, USA) according to the manufacturer's instructions. Modified DNA was amplified by PCR with the outer primers for H19 f: 5′-AGG TGT TTT AGT TTT ATG GAT GAT GG-3′ and r: 5′-TCC TAT AAA TAT CCT ATT CCC AAA TAA CC-3′ [29] (6005–6326 of AF087017; 18 CpG) and for KvDMR f: 5′-TGT TTT TGT AGT TTA TAT GGA AGG GTT AA-3′ and r: 5′-CTC ACC CCT AAA AAC TTA AAA CCT C-3′ [30] (2008-260 bp fragment – 24 CpG 66531-66801 of U90095). PCR was performed using a modified touchdown protocol that consisted of an initial denaturation step at 94°C for 3 min, followed by 4 cycles of 94°C for 40 seconds, 62°C for 40 seconds and 72°C for 45 second. After additional 6 cycles of the same length with 60°C annealing temperature, 20 cycles were performed with successive annealing temperature decrements of 0.5°C in every cycle, followed by 15 cycles with 52°C annealing temperature. The amplified DNA fragments were sub-cloned into pJet1.2/blunt (Fermentas, Glen Burnie, Maryland, USA) for sequencing. Analysis of sequences and diagram generation was performed using BISMA [31].

### Data analysis

Results were expressed as the mean ± SEM. Statistical analysis was performed using the Student *t*-test.

## Results

### Neural differentiation of hpESCs

**Figure 1:**
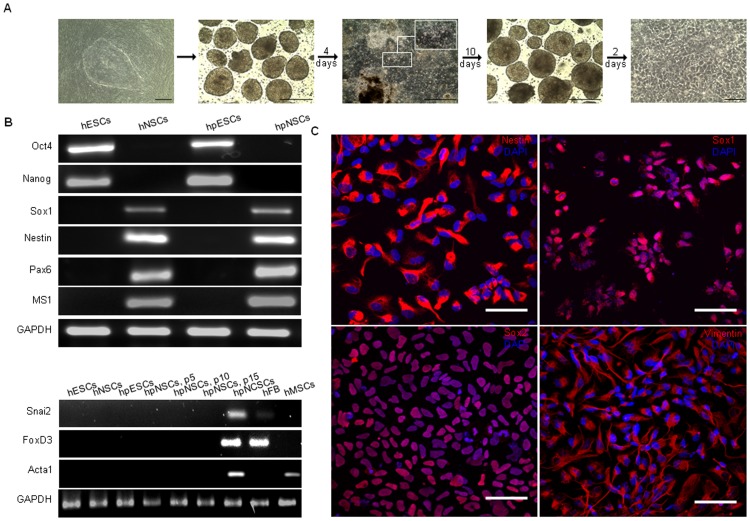
hpESCs generate NSCs. (**A**) Time-lapse, phase-contrast images illustrating the individual stages of neural *in vitro* differentiation of hpESC line LLC9P towards hpNSCs. Starting from hpESCs grown on human foreskin fibroblasts to hpESC-derived floating embryoid bodies, attached embryoid bodies (insert indicates rosette-like structures), floating neurospheres and NSCs. Scale bars, left panel: 0.5 mm; other panels: 0.25 mm. (**B**) RT-PCR analysis for the expression of pluripotency (*Oct4* and *Nanog*) and neural stem cell markers (*Sox1*, *Nestin*, *Pax6* and *MS1*) in undifferentiated hESC and hpESC cultures, and in hNSC and hpNSC cultures. Also shown is a RT-PCR analysis for the expression of the neural crest cell markers *Snai2* and *FoxD3* and the mesodermal marker *Acta1* in undifferentiated hESCs, hNSCs, hpESCs, and hpNSCs at passages 5, 10 and 15. Controls shown are analyses of human parthenogenetic neural crest stem cells (hpNCSCs), human fetal brain (hFB) and human adipose tissue-derived mesenchymal stromal cells (hMSCs). (**C**) Representative confocal images of hpNSC cultures immunostained with antibodies specific for *Nestin*, *Sox1*, *Sox2*, and *Vimentin* are shown. Scale bars: 50 µm; n = 3.

Uniparental hpESCs (ESC lines LLC9P and LLC6P [10]) were cultured using a multi-step *in vitro* differentiation protocol that can produce NSCs from pluripotent stem cells [26]. Initial differentiation of hpESCs produced floating embryoid bodies (EB) that formed neural rosettes after attachment (Fig. 1A, Fig. S1 A). Isolated neural rosettes could be expanded as floating neurospheres that formed monolayers with NSC-like homogeneous morphology after plating onto polyornithine/laminin-coated plates (Fig. 1A right panel). The NSC identity of monolayer cells was confirmed by gene expression analysis revealing upregulation of NSC markers *Sox1*, *Nestin*, *Pax6*, and *Musashi1* (*MS1*) (Fig. 1B,. Fig. S1 B), silencing of pluripotency marker genes (*Oct4* or *Nanog*), and absence of activation of markers of non-neural lineage commitment, including neural crest (*Snai2, FoxD3*) and mesoderm *(Acta1)*. Expression of the neural stem cell markers *Nestin*, *Sox1*, *Sox2* and *Vimentin* in hpNSC cultures was ubiquitous and not limited to subsets of cells (Fig. 1C and in. Fig. S1 C). Upon differentiation, two 10 cm^2^ plate dishes of LLC9P hpESCs yielded a mean of 29 (±3.5) million hpNSCs whereas LLC6P hpESCs generated 11.8 (±1.7) million cells. As a recent report described aberrant expression levels of molecules related to spindle formation and chromosome segregation in hpESCs [20], we verified expression of these markers in undifferentiated LLC6P and LLC9P hpESCs, and detected variations in gene expression not only between PG and N cells but also between individual hpESCs (Fig. S2 A). Additionally, reduced levels of extracellular matrix (ECM) transcripts had been detected in PG compared to N (biparental) ESCs [22]. We observed variation in ECM transcript levels of ECM molecules between individual PG and N cell lines, with lower expression in LLC6P hpESCs compared to LLC9P cells and to hESCs (Fig. S2 B). In conclusion, hpESCs can differentiate into hpNSCs that express neural stem cell markers in the absence of pluripotency or neural crest cell marker expression.

### Terminal differentiation of hpESC-derived hpNSCs

**Figure 2:**
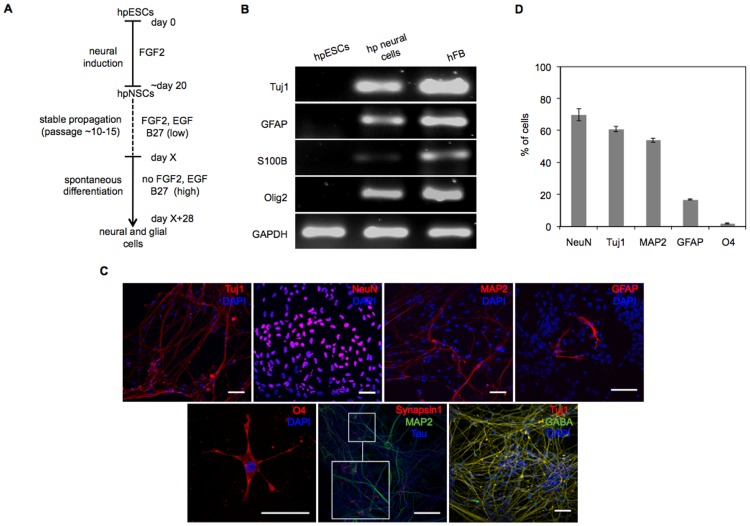
hpNSC differentiation into neuronal and glial cell types. (**A**) Schematic representation of neural *in vitro* differentiation of hpESCs (LLC9P) towards neural subtypes (adapted from [26]). (**B**) RT-PCR analysis of hpESCs, hpNSC-derived neural cell cultures (hp neural cells) and of an hFB sample for the expression of neural transcripts: *Tuj1*, *GFAP*, *S100B* and *Olig2*. (**C**) Immunostainings of hpNSC-derived neural cell cultures with antibodies specific for: *Tuj1, NeuN, MAP2, GFAP, O4, Synapsin1/Map2/Tau* (insert shows higher magnification), *Tuj1*/GABA. Cells were counterstained with DAPI. (**D**) Percentages of neural subtypes after differentiation are given. The percentages were determined by counting neuronal or glial marker- and DAPI-positive cells. ImageJ software was used for counting. Scale bars: 50 µm; n≥3.

To study the neural differentiation potential of hpNSCs (LLC9P), cells were subjected to growth factor withdrawal to induce terminal differentiation (Fig. 2A). In contrast to undifferentiated hpESCs and similar to a human fetal brain isolate, hpNSC-derived cells (differentiated for 28 days) expressed neuronal (*Tuj1* - class III beta-tubulin), astrocyte (*GFAP* - glial fibrillary acidic protein; *S100B* - S100 calcium binding protein B) and oligodendrocyte (*Olig2* -oligodendrocyte transcription factor 2) lineage-specific transcripts (Fig. 2B) and cell type-specific protein markers *Tuj1, NeuN, MAP2* (microtubule-associated protein 2, neurons), *GFAP* (astrocytes) and *O4* (oligodendrocytes) (Fig. 2C). Expression of the presynaptic vesicle protein *Synapsin1*, the dendritic marker *MAP2* and the axonal marker *Tau* was also detectable at this stage of differentiation (Fig. 2C). 95±1.3% of *Tuj1*/DAPI positive cells co-expressed the neurotransmitter GABA (γ-aminobutyric acid) (Fig. 2C). Overall, we observed that hpNSCs favor neuronal differentiation (61±1.6% of cells), whereas glial cells were less frequently detectable (17±0.3% of cells). Oligodendrocytes were detected only after 6 weeks of differentiation (2±0.3% of cells) (Fig. 2D). Similar results of neuronal and astroglial differentiation were observed for the hpESC line LLC6P (. Fig. S3 A, B), with the exception that O4-positive cells were not detected (Fig. S3 C).

**Figure 3:**
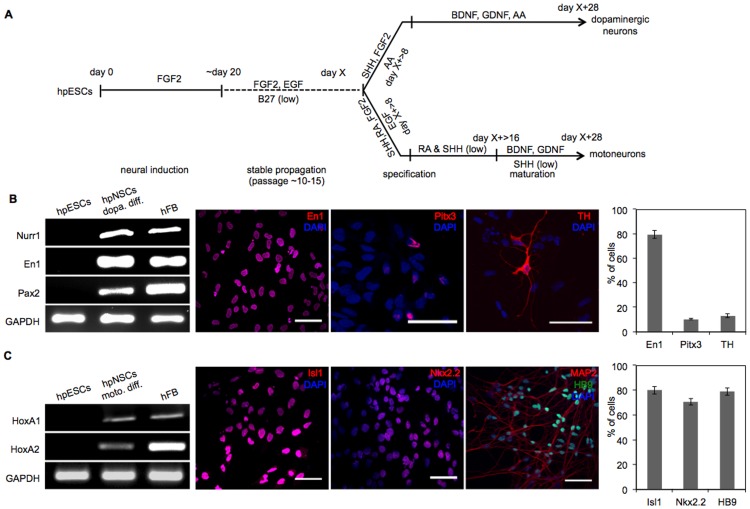
Differentiation of hpNSCs towards dopaminergic neurons and motoneurons. (**A**) Schematic representation of *in vitro* neural differentiation of hpESCs (LLC9P) towards dopaminergic neurons and motoneurons (adapted from [26]). (**B**) RT-PCR analysis for the expression of midbrain transcripts (*Nurr1*, *En1* and *Pax2*). Immunostaining with antibodies specific for *En1*, *Pitx3* and *TH*. Cells were co-stained with DAPI. Percentages of DAPI and *En1*, *Pitx3* or *TH* positive cells are indicated. (**C**) RT-PCR analysis for the expression *HoxA1* and *HoxA2*. Shown are immunostainings with antibodies specific for: *Isl1*, *Nkx2.2* and *MAP2/HB9*. Cells were co-stained with DAPI. Shown are percentages of DAPI and *Isl1*, *Nkx2.2* and *HB9*- and DAPI-positive cells. Scale bars: 50 µm; n = 3.

Next we analyzed whether hpNSCs remain responsive to instructive regionalization cues known to induce dopaminergic [27] or motoneuron differentiations [28]. To induce formation of TH^+^ (tyrosine hydroxylase)-neurons, hpNSCs were first cultured in media supplemented with sonic hedgehog (SHH) and FGF8b, followed by culture in media containing BDNF and GDNF (Fig. 3A, top). After 28 days of differentiation, cells expressed transcripts for the midbrain-specific *Nurr1* (nuclear receptor related 1 protein), *En1* (engrailed homeobox 1) and *Pax2* (paired box gene 2) (Fig. 3B). Immunocytochemical staining verified upregulation of the midbrain markers *En1* and *Pitx3* (paired-like homeodomain 3), which are transcription factors required for differentiation and survival of midbrain dopaminergic neurons, and for *TH* (dopamine biogenesis) (Fig. 3B). We observed 79.8±3.2% *En1*, 10.8±0.6% *Pitx3* and 13.3±1.6% *TH* positive cells (Fig. 3B).

To explore the motoneuron potential of hpNSCs, we exposed cells to sequential growth factor combinations that induce a motoneuron fate (Fig. 3A). After 28 days of differentiation, transcripts of the motoneuron markers *HoxA1* and *HoxA2* were detectable in differentiated cultures but not in undifferentiated hpESCs. Correspondingly, immunostaining revealed nuclear expression of *Isl1* (ISL LIM homeobox1, marker for motoneuron progenitors), *Nkx2.2* (NK2 homeobox 2, ventral brain marker), *HB9* (motor neuron and pancreas homeobox 1, motoneuron marker) and *MAP2* (neuronal marker) (Fig. 3C). hpNSCs generated 80.1±3% *Isl1*, 70.9±2.6% *Nkx2.2* and 79.1±2.9% *HB9* positive cells (Fig. 3C). In summary, these data indicate that hpNSCs are responsive to instructive regionalization cues and that hpNSCs can differentiate into cells that express dopaminergic and motoneuron markers. Neuronal cells that express dopaminergic or motoneuron markers were also observed upon differentiation of hpESC line LLC6P (Fig. S4).

### Electrophysiological analysis of PG neurons

**Figure 4:**
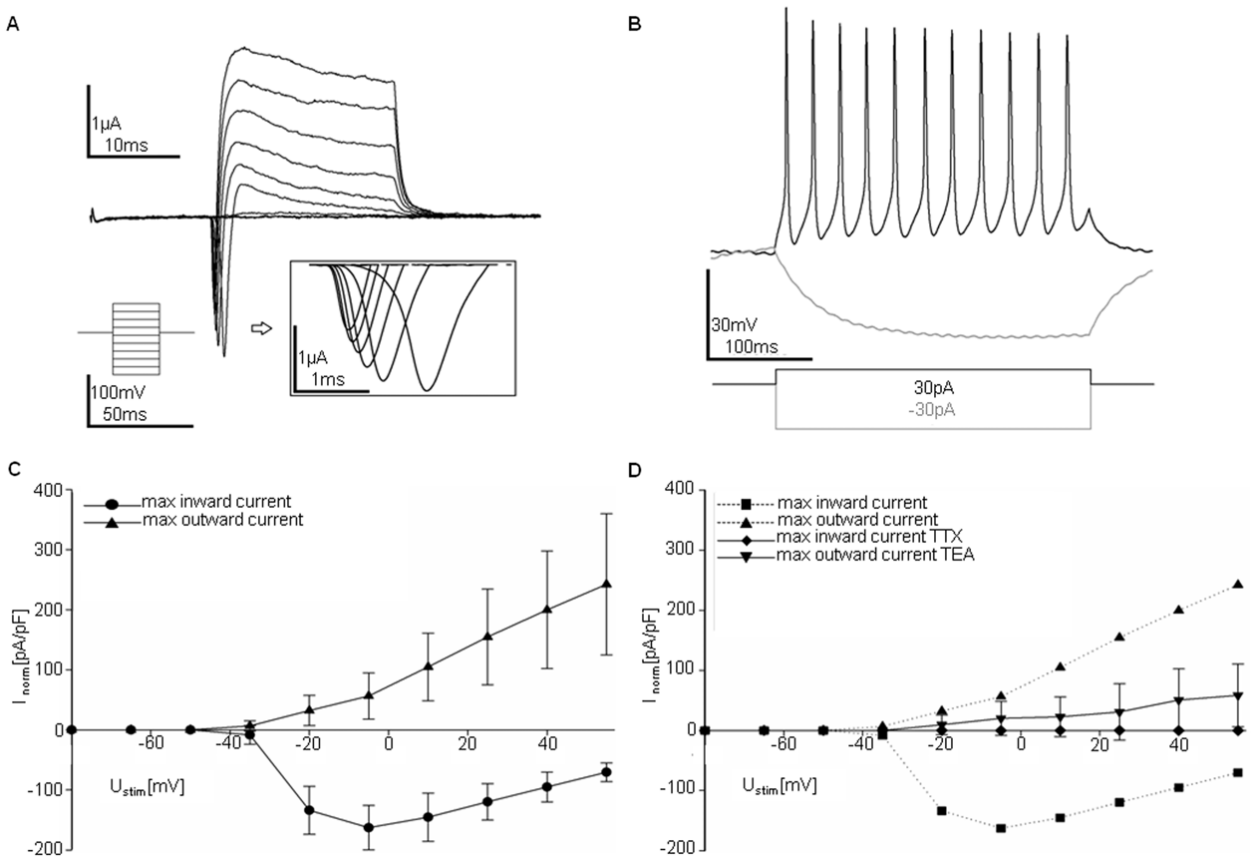
Electrophysiologically active PG neurons. Representative electrophysiological analysis of hpESC-derived neurons (LLC9P) after *in vitro* differentiation for 28 days. (**A**). Representative current traces in whole cell configuration responding to step depolarization. Insert shows sodium currents. Stimulation via stepwise increase of membrane potential (−80 mV to +55 mV, step size 15 mV) in VC-mode. (**B**) Representative traces of membrane potential responding to step depolarization by current injection in CC mode; depolarization - black line, hyperpolarization - grey line. (**C**) Current (I)/voltage (V) curves of voltage clamp (VC)-stimulation. Stimulation potential [mV] is plotted against the highest and the lowest measured current (current is normalized to cell size [pA/pF]). (**D**) I/V curves of VC-stimulation before and after treatment with tetrodotoxin (TTX, sodium channel blocker) or tetraethylamonium (TEA, potassium channel blocker). Stimulation potential [mV] is plotted against the highest and the lowest measured current (current was normalized to cell size [pA/pF]). n = 3.

We further investigated whether PG neurons can functionally mature *in vitro*. As shown in Table S1, electrophysiological properties of PG neurons at 28 days of differentiation were comparable to those reported in literature for human *in vitro* induced neuronal cells [32]. PG neurons showed typical neuronal Na^+^/K^+^ currents in voltage clamp mode (vc stimulation pattern: −80 mV to +55 mV, step size 15 mV, stimulation time 20 ms) (Fig. 4A). Depolarizing step current injections over a 500 ms time period elicited multiple action potentials with a maximum frequency of 30 Hz (Fig. 4B). When maximum in- and outward currents were plotted against the corresponding stimulation voltage, PG neurons depicted a typical neuron-like current pattern (Fig. 4C) that responded to selective pharmacological blockers of sodium (tetrodotoxin) and potassium (tetraethylammonium) channels (Fig. 4D).

### Analysis of imprinted genes

**Figure 5:**
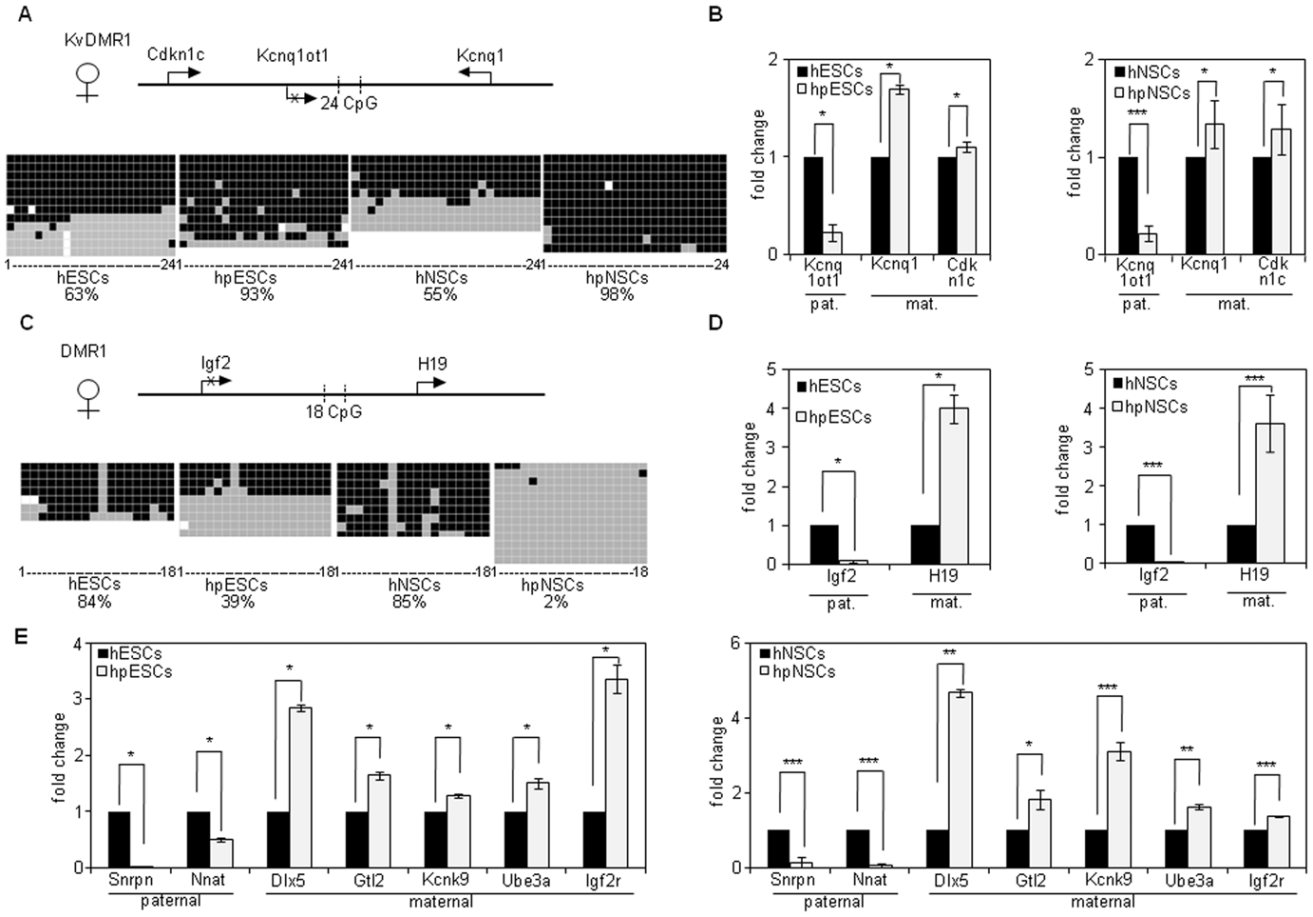
Analysis of the methylation status of differentially methylated regions (DMRs) and expression analysis of imprinted genes. Shown are comparative bisulfite sequencing analysis and analysis of imprinted gene expression in hESCs, hNSCs (I3) and in hpESCs, hpNSCs (LLC9P) respectively. (**A**) Bisulfite sequencing of KvDMR1 (position: 66531–66801) in N and PG cells. The location of KvDMR1 and the transcriptional start sites of *Cdkn1c*, *Kcnq1ot1* and *Kcnq1* are indicated (maternal allele). Black boxes represent methylated CpGs; grey boxes show unmethylated CpGs; white boxes: not analyzed. Percentages of CpG methylation are indicated. (**B**) Shown are RT-PCR analyses of imprinted genes regulated by *Kcnq1ot1* long non-coding RNA in PG and N cells. The relative expression represents the fold change of gene expression in PG compared to N cells, respectively. Fold change was calculated by the 2^−ΔΔCt^ method. The housekeeping gene GAPDH was used as a reference gene. Expression levels of N cells were set to 1. n = 3. (**C**) Shown are the location of DMR1 (position: 66531–66801), gene regions of Igf2 and H19 (maternal allel) and results of bisulfite sequencing analyses of DMR1. (**D**) Shown are gene expression analyses of *Igf2* and *H19* in N and PG cells. n = 3. (**E**) Expression analysis of imprinted genes of other loci by quantitative RT-PCR. n = 3, * p<0.05, ** p<0.01, *** p<0.001 by Student's *t*-test.

To assess the status of epigenetic marks involved in the control of imprinted gene expression during neural differentiation of hpESCs, we analyzed the methylation status of CpG islands of two differentially methylated regions, the 5′ region of the long non-coding RNA *Kcnq1ot1* (KvDMR1) and the H19 DMR1 (Fig. 5). Methylation of KvDMR1 on the maternal allele, acquired during germ cell development, is associated with silencing of *Kcnq1ot1*, whereas *Kcnq1ot1* expression from the unmethylated paternal allele is involved in domain-wide chromatin repression of a cluster of genes including *Cdkn1c* and *Kcnq1* [33]. Consistent with PG origin, CpGs of the KvDMR1 were mostly methylated in hpESCs and hpNSCs, while conventional hESCs and hNSCs exhibited 50% methylation, indicating the presence of maternal and paternal alleles (Fig. 5A). Quantitative RT-PCR analysis revealed absence of *Kcnq1ot1* RNA in hpESCs and hpNSCs, and higher expression of *Kcnq1* but not *Cdkn1c* in PG compared to N cells (Fig. 5B).

Differential germline-acquired methylation of the H19 DMR, a chromatin insulator, controls reciprocal allelic silencing of the *Igf2* and *H19* genes. On the unmethylated maternal allele, CTCF binding blocks enhancer-initiated transcription of *Igf2*, allowing *H19* expression, while methylation on the paternal allele abolishes insulator function permitting *Igf2* expression and leading to silencing of *H19* in *cis* [34]. The majority of DMR1 CpGs were methylated in N cells, whereas PG cells exhibited partial or complete absence of CpG methylation (Fig. 5C). Parent of origin-specific expression of *Igf2* and *H19* was maintained in PG cells with absence of *Igf2* expression and overexpression of *H19* in PG compared to N cells (Fig. 5D).

Expression analyses of additional imprinted genes revealed that silencing of the paternally expressed *Snrpn* and *Nnat* genes was preserved in hpESCs and hpNSCs. Levels of the maternally expressed *Gtl2*, *Dlx5* and *Kcnk9* genes were overall higher in hpESC and hpNSC compared to N cells. However, *Igf2r* expression was elevated only in hpESCs but not in hpNSCs (Fig. 5E, Fig S5). Together, parent of origin-specific gene expression control appears to be largely maintained in hpESC lines LLC6P and LLC9P, and neural differentiation is not associated with a loss of silencing of paternally expressed genes that were analyzed.

## Discussion

Our objective was to define the neural differentiation potential of hpESCs *in vitro*. In summary, we describe that hpESCs - despite having a maternal genome only - generate proliferating NSCs that are capable of differentiating towards neurons that express specific markers for neuronal transmitters and synaptic proteins and show electrical activity. PG cells maintain allele-specific expression of imprinted genes.

Our results confirm the neural differentiation potential of hpESCs. In particular, the results show that hpESCs can generate proliferating hpNSCs, which can further differentiate not only to early neural lineages as described earlier [8]–[10] but also into mature neural and glial cell types. hpNSCs respond to signals directing the derivation of ventral midbrain dopaminergic and motoneurons. Similar to earlier reports on conventional NSCs, we observed high frequencies of GABAergic neurons [26], [35], [36]. The reason for the preference of hpNSCs towards GABAergic differentiation is not clear, although the high concentrations of mitogens present during the expansion of hpNSCs are likely a contributing factor. The preference towards neuronal and less glial differentiation outcomes mimics the developmental potential of NSCs in the developing brain [37]. We further show that PG neuron-like cells were capable to generate action potentials and possessed membrane characteristics similar to newly formed neurons [26], [35]. The unperturbed neural differentiation potential of hpESCs is consistent with earlier reports of successful murine AG ESC-derived neurogenesis [11], [12], [16]. Our analyses indicate that uniparental ESCs are less restricted in their neural developmental potential than predicted from *in vivo* studies [18], [38].

Previous analyses of neural differentiation potential of hpESC via sphere formation suggested that hpESCs yielded low quantities and less mature neural cells compared to conventional ESCs [22]. Our results are in contrast to this report, with several factors likely to contribute to such a difference. Firstly, we subjected hpESC lines (a subset of those used by [22]) to an alternative differentiation protocol, which was optimized towards the derivation of a homogeneous population of NSCs, and minimized spontaneous differentiation and lineage restriction. Secondly, our results revealed differences in gene expression of extracellular matrix proteins not only between hpESCs and hESCs but also between individual hpESC cell lines. Therefore, low yields of ES-derived NSCs from LLC6P compared to LLC9P hpESCs may be related to poor cell-cell interaction caused by low levels of ECM gene expression in LLC6P [22]. High hpNSC yields of LLC9P hpESCs could be caused by the elevated expression of the early neuroblast marker *NCAM1* [39]. We also observed differences in the expression of mitotic checkpoint genes in hpESCs in comparison to hESCs as well as between the two hpESC cell lines. Possible explanations for these differences likely include cell line to cell line variation [40] and potentially an underlying genetic instability of uniparental ESCs [20]. A recent report suggested that PG ES cells of different species origin show centrosomal amplification and chromosomal instability [41]. Previous analyses of hpESC line LLC6P and LLC9P revealed a normal human 46,XX karyotype suggesting that the cells under study are chromosomally normal [10].

ESC lines can undergo epigenetic changes during *in vitro* culture [42]–[44]. Although hESC exhibit a substantial degree of epigenetic stability, despite differences in genetic background, derivation and expansion conditions [44], imprinted loci have been found to exhibit varying susceptibility to culture-induced epigenetic changes, with more stability at the Kcnq1ot1 locus and less at H19/Ifg2 [43]. Consistent with such observations, we detected conservation of maternal-specific CpG methylation at the KvDMR, low expression of *Kcnq1ot1*, and upregulation of maternally expressed *Kcnq1* in PG cells, although *Cdkn1c* transcripts were only upregulated in one PG cell line (LLC6P). Our analyses of methylation of DMR1 of the *H19/Igf2* locus agree with previous reports suggesting that late passage hESCs are prone to hypermethylation this region [43]. Here we observe hypermethylation in hESCs and hNSCs, and modest gain of CpG methylation in hpESCs. Despite these changes, *Igf2* and *H19* transcript levels in hpESCs and NSCs remained consistent with PG origin, indicating that regulatory mechanisms other than CpG methylation are involved in imprinting control of *H19* and *Igf2* [45]. Other paternal (*Snrpn* and *Nnat*) and maternal (*Dlx5*, *Gtl2*, *Ube3a* and *Kcnk9*) imprinted genes maintained their parent of origin-specific gene expression pattern. *Igf2r* expression was elevated only in hpESCs but not in hpNSCs. The molecular basis for this remains unclear. Increased methylation in the higher passage hESCs used in our study may be a consequence of the *in vitro* expansion of ESCs, however, overall, our analyses indicate that PG cells are epigenetically as stable as N cells.

Considering the putative prevalence of imprinted genes expressed in the developing mammalian brain [17] and the altered expression of ECM genes and molecules related to spindle formation and chromosome segregation [20], [22], the capacity of hpESCs to undergo similar *in vitro* neural differentiation as hESCs seems surprising. This suggests either that there is a less stringent role for imprinted gene expression during neuronal *in vitro* differentiation, or that a requirement for balanced expression of imprinted genes is not required for differentiation to the stages analyzed. While we show the successful *in vitro* differentiation of hpESCs into neural subtypes and that PG neurons develop synaptic contacts and electrical activity, transplant models will ultimately be required to assess the broader neural differentiation potential of hpESCs.

## Supporting Information

Figure S1 **hpESC-derived hpNSCs (hpESC line LLC6P).** (**A**) Images of individual differentiation stages during the derivation of hpNSCs. hpESCs, floating embryoid bodies, attached embryoid bodies which exhibit rosette-like structures, floating neurospheres and hpNSCs. Scale bars, left panel: 0.5 mm; other panels: 0.25 mm. (**B**) Expression of *Oct4*, *Nanog*, *Sox1*, *Nestin*, *Pax6* and *MS1* in hESCs, hNSCs, hpESCs, and hpNSCs by RT-PCR. GAPDH is the house-keeping control. (**C**) Immunostaining of hpESC-derived hpNSCs for *Nestin*, *Sox1*, *Sox2* and *Vimentin* expression. Nuclei were counterstained with DAPI. Confocal images of a representative analysis are shown. Scale bars: 50 µm; n = 3.
(TIF)

Figure S2 **Expression analysis of mitotic checkpoint and extracellular matrix genes by RT-PCR.** (**A**) Expression level in PG (LLC9P and LLC6P) and N (I3 and H9) ESCs were analyzed by RT-PCR. The genes analyzed are mitotic arrest deficient 1 (*MAD1*), budding uninhibited by benzimidazoles 1 (*BUB1*), centromere protein E (*CENPE*), *TTK kinase* (human homologue of the yeast monopolar spindle 1 kinase), *aurora A kinase*, Myc-associated factor X (*MAX*), SWI-Independent 3 (*SIN3*). (**B**) RT-PCR expression analysis of extracellular matrix molecules: matrix metalloproteinase 1 (*MMP1*), matrix metalloproteinase 7 (*MMP7*), collagen type XI alpha 1 (*COL11A1*), neural cell adhesion molecule 1 (*NCAM1*), vascular cell adhesion molecule 1 (*VCAM1*) and integrin alpha-8 (*ITGA8*) in hpESCs (LLC9P and LLC6P) compared to hESCs (I3 and H9). Expression levels of N cells were set to 1. Fold change was calculated by the 2^−ΔΔCt^ method. The housekeeping gene GAPDH was used as a reference. n = 3, * p<0.05, ** p<0.01, *** p<0.001 by Student's *t*-test.
(TIF)

Figure S3 **In vitro differentiation of hpNSCs into neural subtypes (LLC6P).** (**A**) Expression of neuronal and glial markers *Tuj1*, *GFAP*, *S100B*, *Olig2* and the house-keeping gene *GAPDH* by RT-PCR. (**B**) hpNSC-derived neuronal and glial cells were stained with antibodies specific for: *Tuj1*, *NeuN*, *MAP2*, *GFAP*, *GABA*, *Synapsin1* or *Tau*. The nuclear stain DAPI was used. n = 4. (**C**) Percentages of immuno-reactive neuronal and glial subtypes are given. Scale bars: 50 µm; n≥4.
(TIF)

Figure S4 **Differentiation of hpNSCs towards dopaminergic and motoneurons (LLC6P).** (**A**) RT-PCR analysis for expression of *Nurr1*, *En1*, *Pax2* by RT-PCR. Immunostainings for expression of dopaminergic neuron-specific markers: *En1*, *Pitx3* and *TH*. Percentage of cells immunostained for *En1*, *Pitx3*, *TH* and co-stained with DAPI. (**B**) Expression of *HoxA1* and *HoxA2* analyzed by RT-PCR. Immunostainings for expression of motoneuron markers: *Isl1*, *Nkx2.2* and *HB9*. Nuclei were counterstained with DAPI. Percentage cell counts of *Isl1*, *Nkx2.2* and *HB9*- and DAPI-positive cells are indicated. Scale bars: 50 µm; n = 3.
(TIF)

Figure S5 **RT-PCR analysis of imprinted genes in hpESCs and hpNSCs (LLC6P).** Relative expression levels of the imprinted genes: *Ifg2, Snrpn Nnat* and *Kcnq1ot1* (paternally expressed) and, *Dlx5*, *H19*, *Ube3a*, *Igf2r*, *Kcnq1*, *Cdkn1c*, *Gtl2* and *Kcnk9* (maternally expressed) were analyzed by RT-PCR in PG and N cells (I3 and H9). The 2^−ΔΔCt^ method was used to calculate fold change in the expression of imprinted genes. Expression levels N cells were set to 1. GAPDH was used as a reference gene. n = 3, * p<0.05, ** p<0.01, *** p<0.001 by Student's *t*-test.
(TIF)

Table S1 **Electrophysiological characteristics of PG neurons.**
(DOCX)
