# Supplementary figures and images for: Functional Neuronal Cells Generated by Human Parthenogenetic Stem Cells
Source: PLoS One. 2012 Aug 6;7(8):e42800. doi: 10.1371/journal.pone.0042800 (PMC3412801; doi:10.1371/journal.pone.0042800)

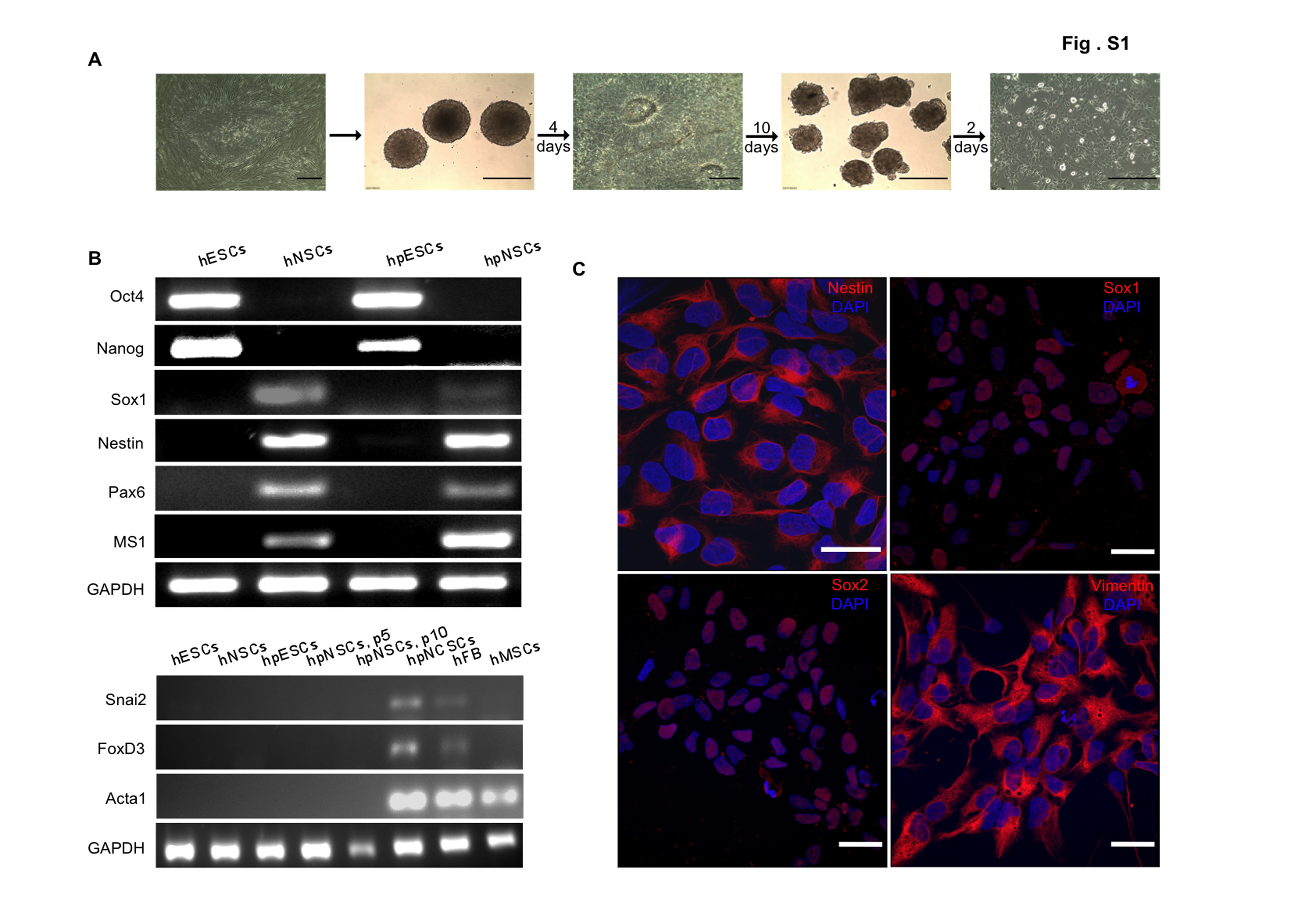

Supplement: Figure S1 — hpESC-derived hpNSCs (hpESC line LLC6P). (A) Images of individual differentiation stages during the derivation of hpNSCs. hpESCs, floating embryoid bodies, attached embryoid bodies which exhibit rosette-like structures, floating neurospheres and hpNSCs. Scale bars, left panel: 0.5 mm; other panels: 0.25 mm. (B) Expression of Oct4, Nanog, Sox1, Nestin, Pax6 and MS1 in hESCs, hNSCs, hpESCs, and hpNSCs by RT-PCR. GAPDH is the house-keeping control. (C) Immunostaining of hpESC-derived hpNSCs for Nestin, Sox1, Sox2 and Vimentin expression. Nuclei were counterstained with DAPI. Confocal images of a representative analysis are shown. Scale bars: 50 µm; n = 3. (TIF) [file pone.0042800.s001.tif]

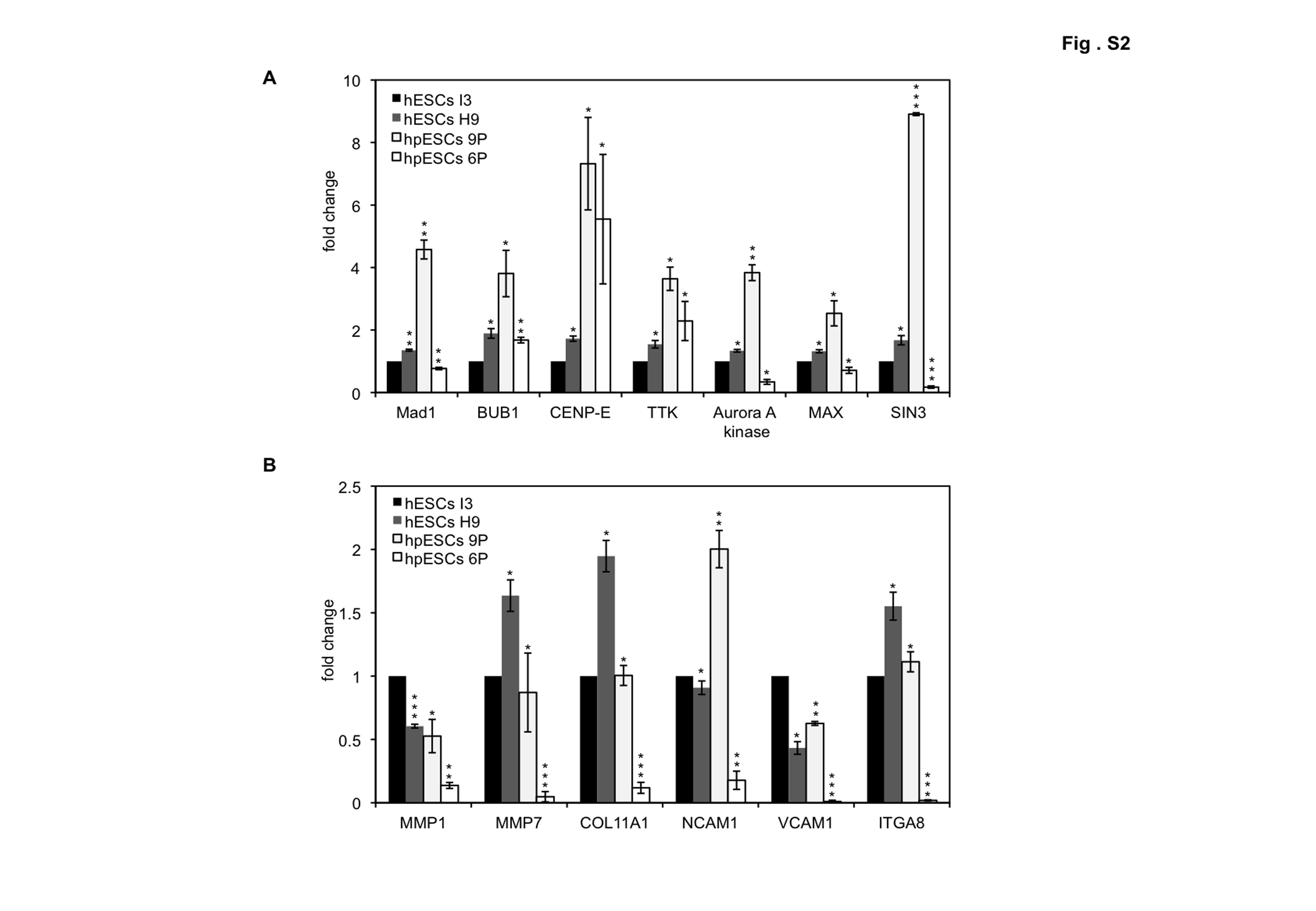

Supplement: Figure S2 — Expression analysis of mitotic checkpoint and extracellular matrix genes by RT-PCR. (A) Expression level in PG (LLC9P and LLC6P) and N (I3 and H9) ESCs were analyzed by RT-PCR. The genes analyzed are mitotic arrest deficient 1 (MAD1), budding uninhibited by benzimidazoles 1 (BUB1), centromere protein E (CENPE), TTK kinase (human homologue of the yeast monopolar spindle 1 kinase), aurora A kinase, Myc-associated factor X (MAX), SWI-Independent 3 (SIN3). (B) RT-PCR expression analysis of extracellular matrix molecules: matrix metalloproteinase 1 (MMP1), matrix metalloproteinase 7 (MMP7), collagen type XI alpha 1 (COL11A1), neural cell adhesion molecule 1 (NCAM1), vascular cell adhesion molecule 1 (VCAM1) and integrin alpha-8 (ITGA8) in hpESCs (LLC9P and LLC6P) compared to hESCs (I3 and H9). Expression levels of N cells were set to 1. Fold change was calculated by the 2−ΔΔCt method. The housekeeping gene GAPDH was used as a reference. n = 3, * p<0.05, ** p<0.01, *** p<0.001 by Student's t-test. (TIF) [file pone.0042800.s002.tif]

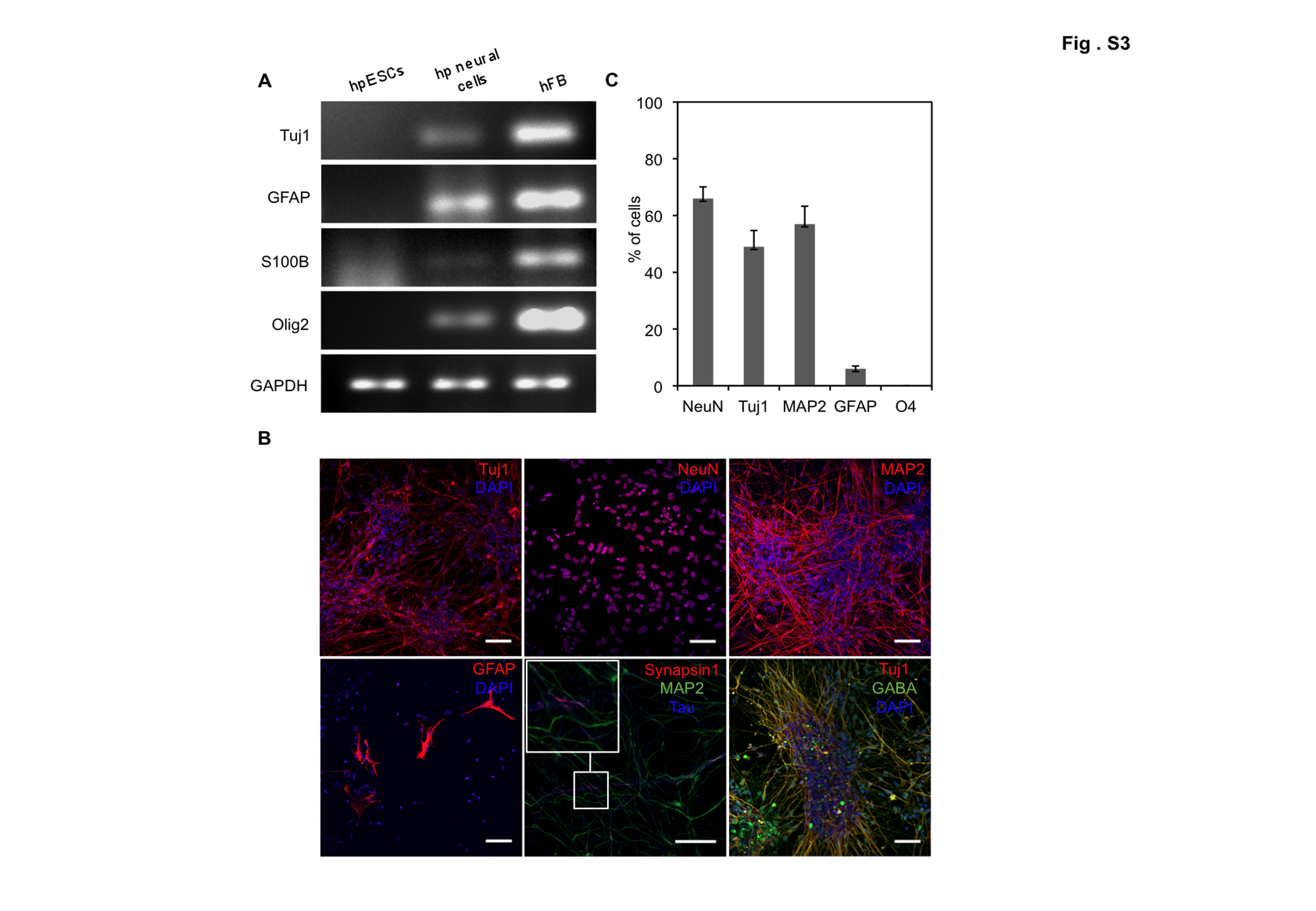

Supplement: Figure S3 — In vitro differentiation of hpNSCs into neural subtypes (LLC6P). (A) Expression of neuronal and glial markers Tuj1, GFAP, S100B, Olig2 and the house-keeping gene GAPDH by RT-PCR. (B) hpNSC-derived neuronal and glial cells were stained with antibodies specific for: Tuj1, NeuN, MAP2, GFAP, GABA, Synapsin1 or Tau. The nuclear stain DAPI was used. n = 4. (C) Percentages of immuno-reactive neuronal and glial subtypes are given. Scale bars: 50 µm; n≥4. (TIF) [file pone.0042800.s003.tif]

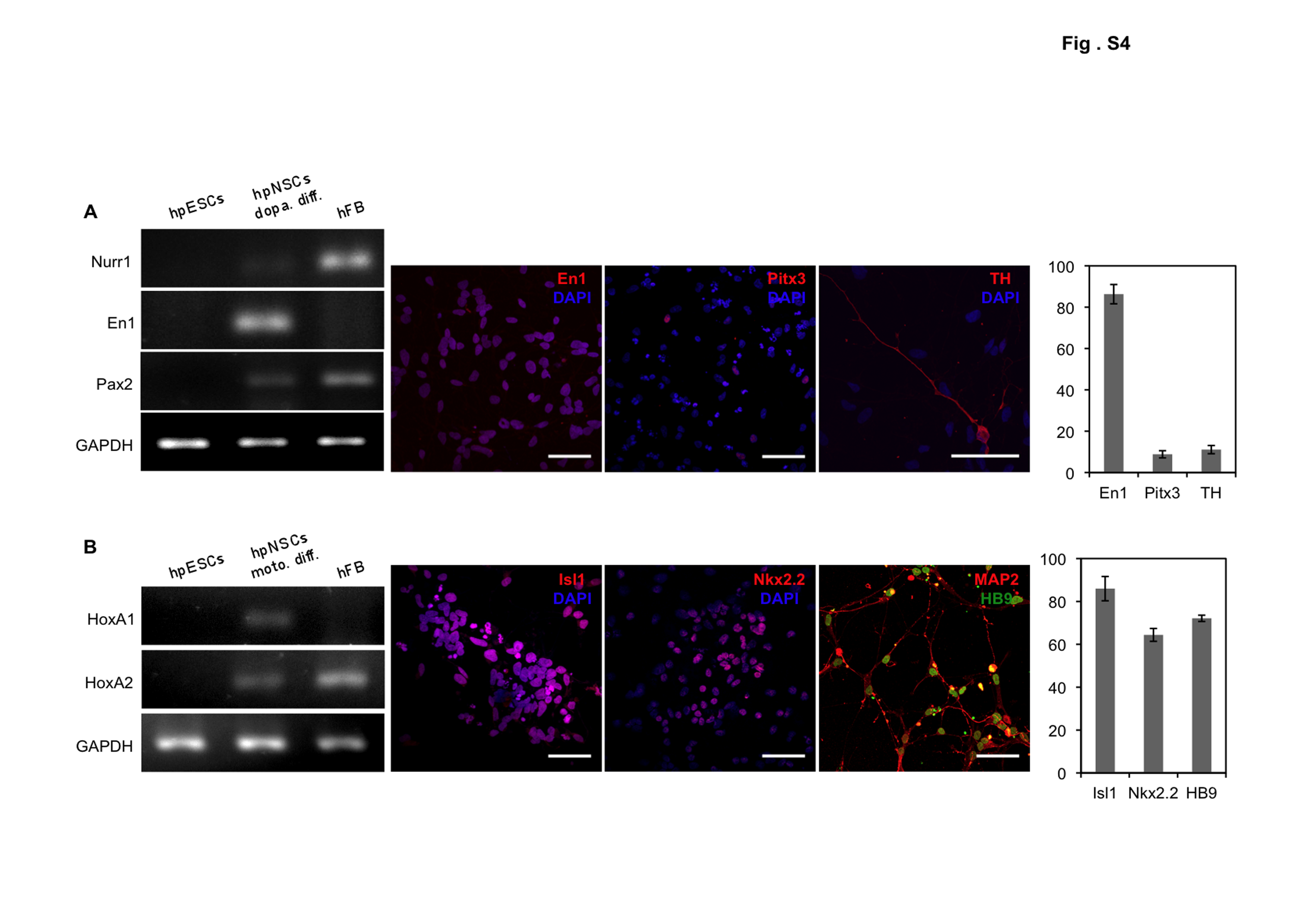

Supplement: Figure S4 — Differentiation of hpNSCs towards dopaminergic and motoneurons (LLC6P). (A) RT-PCR analysis for expression of Nurr1, En1, Pax2 by RT-PCR. Immunostainings for expression of dopaminergic neuron-specific markers: En1, Pitx3 and TH. Percentage of cells immunostained for En1, Pitx3, TH and co-stained with DAPI. (B) Expression of HoxA1 and HoxA2 analyzed by RT-PCR. Immunostainings for expression of motoneuron markers: Isl1, Nkx2.2 and HB9. Nuclei were counterstained with DAPI. Percentage cell counts of Isl1, Nkx2.2 and HB9- and DAPI-positive cells are indicated. Scale bars: 50 µm; n = 3. (TIF) [file pone.0042800.s004.tif]

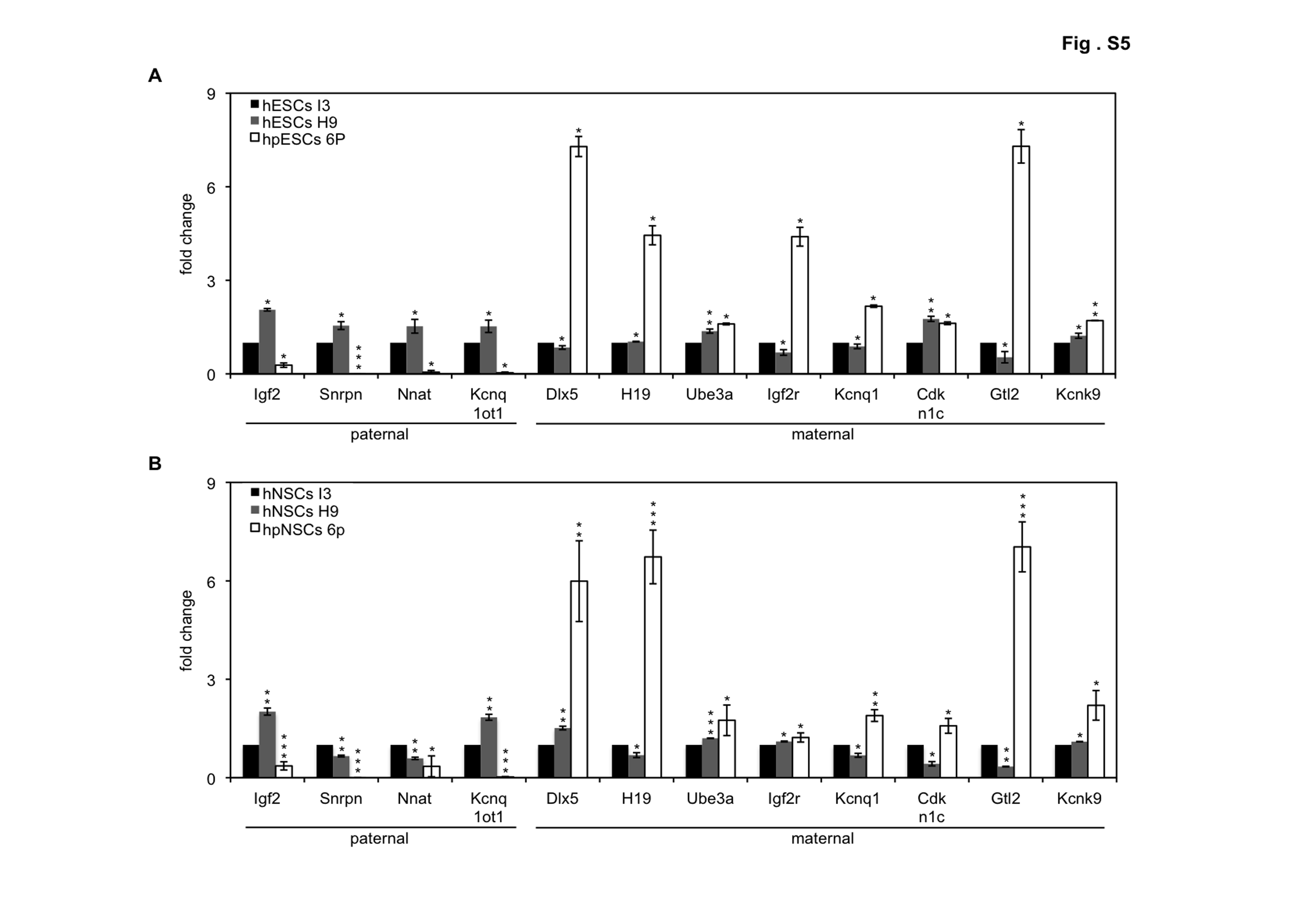

Supplement: Figure S5 — RT-PCR analysis of imprinted genes in hpESCs and hpNSCs (LLC6P). Relative expression levels of the imprinted genes: Ifg2, Snrpn Nnat and Kcnq1ot1 (paternally expressed) and, Dlx5, H19, Ube3a, Igf2r, Kcnq1, Cdkn1c, Gtl2 and Kcnk9 (maternally expressed) were analyzed by RT-PCR in PG and N cells (I3 and H9). The 2−ΔΔCt method was used to calculate fold change in the expression of imprinted genes. Expression levels N cells were set to 1. GAPDH was used as a reference gene. n = 3, * p<0.05, ** p<0.01, *** p<0.001 by Student's t-test. (TIF) [file pone.0042800.s005.tif]
