# Supplementary material for: Functional Neuronal Cells Generated by Human Parthenogenetic Stem Cells
Source: PLoS One. 2012 Aug 6;7(8):e42800. doi: 10.1371/journal.pone.0042800 (PMC3412801; doi:10.1371/journal.pone.0042800)
Supplement: Table S1 — Electrophysiological characteristics of PG neurons. (DOCX) [file pone.0042800.s006.docx]

***Supplemental Table I.*** Electrophysiological characteristics of PG neurons

|  | **mean** | **SD** | **N** |
| --- | --- | --- | --- |
| resting membrane potential (mV) | -32.50 | ± 7.27 | 14 |
| membrane capacitance (pF) | 9.31 | ± 3.88 | 14 |
| series resistance (MΩ) | 7.83 | ± 2.91 | 14 |
| number of induced action potentials | 12 out of 12 recordings |  |  |
